# Maintenance of glutathione levels and its importance in epigenetic regulation

**DOI:** 10.3389/fphar.2014.00088

**Published:** 2014-05-05

**Authors:** José L. García-Giménez, Federico V. Pallardó

**Affiliations:** ^1^Center for Biomedical Network Research on Rare DiseasesValencia, Spain; ^2^INCLIVA Biomedical Research InstituteValencia, Spain; ^3^Department of Physiology, School of Medicine and Dentistry, Universitat de ValenciaValencia, Spain

**Keywords:** DNA methylation, epigenetic regulation, glutathione, S-adenosyl methionine, prodrugs, mental disorders, glutathionylation

Glutathione (GSH) is present in almost all cell types playing an important function in organisms. It is the main antioxidant in many cell types and it also regulates the function of proteins, including transcription factors (reviewed in Pallardó et al., 2009; Markovic et al., 2010; García-Giménez et al., 2013a).

Over recent years, growing evidence has suggested a link between GSH metabolism and the control of epigenetic mechanisms. Epigenetics is defined as the mitotically/meiotically heritable changes in gene expression that are not due to changes in the primary DNA sequence. This link between GSH and epigenetics occurs at different levels. Hence, GSH can affect DNA and histone methylation, and also it binds to histones throughout the cysteines in the histone H3. Pioneer work showed how oxidized GSH inhibits the activity of S-adenosyl methionine synthetase, MAT1A. This key enzyme is involved in the synthesis of S-adenosyl methionine (SAM), which is used by DNA methyltransferases (DNMTs) and histone methyltransferases (HMTs) as a substrate for DNA and histone methylation, respectively (Pajares et al., 1992; Martinez-Chantar and Pajares, 1996). Therefore, it is possible to alter the methylation status of the genome and modify the epigenetic signature in cells by modulating SAM levels. Methylation of DNA and histone constitutes one of the most studied chemical modifications in the epigenetic code, which shapes gene-expression patterns usually, but not always, repressing gene transcription. Interestingly, replenishment of GSH levels recovers the activity of MAT1A (Mato et al., 2002) thereby contributing to the homeostasis of DNA and histone methylation.

GSH influence on the epigenetic mechanisms may go beyond mere regulation of SAM levels by the mechanism described above. Our recent results (García-Giménez et al., 2013b) report the implication of GSH in the regulation of epigenetic mechanisms by redox phenomena. We recently described the S-glutathionylation of histone H3 as a new post-translational modification, PTM in the histone code. In this modification, GSH binds to Cys110 in histone H3 (Histone H3 variants, H3.1, H3.2, and H3.3) producing changes in the stability of the nucleosomes and altering the chromatin structure by decreasing the proportion of α-helices. Interestingly, we also observed how S-glutathionylation of H3 increased in proliferating cells but not in quiescent cells, suggesting that GSH modifies the structure of the chromatin during cell proliferation. The implications of these results are relevant because the opening of the chromatin by histone glutathionylation contributes to the binding of the replication machinery to the DNA. Furthermore, the ability of GSH to open the chromatin may increase the susceptibility of DNA to the attack of DNA-interacting drugs. Our results agree with those obtained by de Luca et al. in which glutathionylation of H3 may facilitate the interaction of doxorubicin with DNA, thereby increasing the effect of the treatment to doxorubicin-resistant MCF7 breast cancer cells (de Luca et al., 2011).

However, the epigenetic mechanisms described above are just two examples of the many epigenetic events in which GSH may be involved. For example, oxidative stress induces the formation of methionine sulfoxide from methionine and this molecule can immediately react with radical hydroxyl to generate a methyl radical that non-enzymatically and non-specifically methylates cytosine in the DNA (Kawai et al., 2010). This phenomenon could produce deleterious effects in our epigenome (Lewandowska and Bartoszek, 2011). Fortunately, a GSH-dependent enzymatic mechanism that prevents the production of methionine sulfoxide does exist. The GSH/glutaredoxin system can regenerate the activated form of methionine sulfoxide reductase, MSR, the enzyme that converts methionine sulfoxide to methionine (Kim, 2012). Therefore, GSH/glutaredoxin/MSR prevents the generation of the methyl radical and contributes to the regeneration of methionine, which in turn is introduced in the methionine cycle to recover the SAM levels.

Furthermore, it was recently reported that oxidative stress affects methionine synthase (MS), an important enzyme in the regeneration of methionine from homocysteine (Muratore et al., 2013). A decrease in MS activity results in elevated levels of homocysteine, which is associated with blindness, neurological symptoms, and birth defects including encephalopathy and megaloblastic anemia (Outteryck et al., 2012). For this reason, cellular redox control in which GSH, as the main cellular antioxidant is involved, confers GSH the relevant role of controlling key enzymatic processes. These are directly related to the homeostasis of DNA methylation to prevent extensive DNA and histone methylation changes, which in turn would produce harmful consequences.

A number of diseases have been described in which impaired methylation and changes in GSH levels have been reported. This is the case of several psychiatric disorders, like schizophrenia (Regland et al., 1994; Yao et al., 2006), bipolar disorder (Frey et al., 2007; Kuratomi et al., 2008), and autism (James et al., 2004, 2006) which underscores the role of GSH and DNA methylation in these diseases.

Therefore, the evidence reported in this manuscript indicates that pharmacological interventions designed to recover GSH homeostasis favor the correct function of the methionine cycle (MAT1A and MS), the replenishment of SAM, and prevent the non-specific methylation reactions by reducing MSR. Furthermore, as mentioned above, oxidative stress controls the activity of MS, which produces methionine. This amino acid is then used by the MAT1A (which is also controlled by the GSH/GSSG ratio) to produce SAM. This intermediate, besides participating as a substrate for DNMTs and HMTs, is also one of the precursors of the transulfuration pathway that finally produces cysteine, which is used for the synthesis of GSH.

Consequently, efforts to increase cellular GSH concentration by direct administration of GSH analogs have been a challenge over recent decades (reviewed in Wu and Batist, 2013). Some approaches were developed for clinical application in order to increase the stability and uptake of GSH by cells (Sheh et al., 1990; Shibata et al., 1995). Therefore, by controlling the levels of both metabolites we can counterbalance the cellular redox and methylation status. In so doing, it was found that the administration of drugs (i.e., butanedisulphonate) that increase the levels of SAM produces the recovery of GSH levels. This strategy was proposed as a therapeutic approach in HIV patients with central nervous system affectation who presented decreased concentrations of SAM and GSH (Castagna et al., 1995). However, the recovery of GSH concentration in human tissues is difficult due to its biochemical and pharmacokinetic properties but different strategies are being developed to replenish GSH levels (for a review see Cacciatore et al., 2010). Due to the short half-life of GSH in human plasma and its difficulty to penetrate the plasma membrane, it is necessary to provide a high dose to recover physiological levels of the GSH. The use of prodrugs is the most promising approach (Anderson and Luo, 1998) and they present the possibility to be used as modulators of GSH and SAM levels in this kind of diseases.

Another possible approach is the use of co-drugs. Two different compounds are administered simultaneously, when using this class of chemicals. Both compounds are linked to each other by using a covalent bond that is cleavable which regenerates the original drug with adequate bioavailability. Investigations in this type of compounds have already been reported (Ehrlich et al., 2007). In addition, as we have described, oxidative stress produces methionine sulfoxide, which is epigenetically toxic. Therefore, the use of antioxidants could prevent the formation of this oxidized amino acid. It was recently reported that antioxidants like vitamin C regulate epigenetic mechanisms. It has been described how vitamin C, modulates the activity of Tet methylcytosine dioxygenases, which are involved in the formation of 5-hydroxymethylcytosine, thus participating in the demethylation of DNA (Chen et al., 2013; Minor et al., 2013). These authors described how vitamin C by acting as a cofactor for Tet proteins enhances the 5-hidroxymethylation of cytosine (5-hmC) at critical loci for somatic cell reprogramming (Chen et al., 2013; Minor et al., 2013). Thus, they described for first time the role of ascorbate modulating the epigenetic control of genome. The authors also studied the effect of GSH as reducing agent, but they observed that levels of 5-hmC were not affected (Minor et al., 2013). However, in their experiments the authors used GSH, which do not penetrate the plasma membrane. It would be very relevant to reproduce their experiments by incubating MEFs with glutathione ethyl ester, which is able to cross the cellular membranes to conclude if GSH can affect the levels of 5-hmC in the DNA.

The related mechanisms described in this manuscript place the GSH and redox control in the landscape of the epigenetic regulation. The modulation of GSH and SAM levels has the potential to control oxidative stress and epigenetics. Therefore, the design of strategies and the synthesis of therapeutic drugs that help to reestablish the levels of these key molecules are crucial for therapeutic interventions in epigenetic-related diseases.

## Conflict of interest statement

The authors declare that the research was conducted in the absence of any commercial or financial relationships that could be construed as a potential conflict of interest.
